# Antibody-based *in vivo* leukocyte label for two-photon brain imaging in mice

**DOI:** 10.1117/1.NPh.9.3.031917

**Published:** 2022-05-24

**Authors:** Lila D. Faulhaber, Olivia D’Costa, Andy Y. Shih, Juliane Gust

**Affiliations:** a Center for Developmental Biology and Regenerative Medicine, Seattle, Washington, United States; b Seattle Children’s Research Institute, Center for Integrative Brain Research, Seattle, Washington, United States; c University of Washington, Department of Pediatrics, Seattle, Washington, United States; d University of Washington, Department of Bioengineering, Seattle, Washington, United States; e University of Washington, Department of Neurology, Seattle, Washington, United States

**Keywords:** leukocyte, CD45, two-photon microscopy, stroke, neurovascular unit

## Abstract

**Significance:**

To study leukocyte-endothelial interactions in a living system, robust and specific leukocyte labeling techniques are needed for *in vivo* two-photon microscopy of the cerebral microvasculature.

**Aim:**

We tested fluorophore-conjugated anti-CD45.2 monoclonal antibodies (mAb) to optimize dosing and two-photon imaging parameters for leukocyte labeling in healthy mice and a venous microstroke model.

**Approach:**

We retro-orbitally injected anti-CD45.2 mAb at 0.04, 0.4, and 2  mg/kg into BALB/c mice and used flow cytometry to analyze antibody saturation. Leukocyte labeling in the cortical microvasculature was examined by two-photon imaging. We also tested the application of CD45.2 mAb in a pathological leukocyte-endothelial adhesion model by photothrombotically occluding cortical penetrating venules.

**Results:**

We found that 0.4  mg/kg of anti-CD45.2 antibody intravenously was sufficient to label 95% of circulating leukocytes. There was no depletion of circulating leukocytes after 24 h at the dosages tested. Labeled leukocytes could be observed as deep as 550  μm from the cortical surface. The antibody reliably labeled rolling, crawling, and adherent leukocytes in venules around the stroke-affected tissues.

**Conclusion:**

We show that the anti-CD45.2 mAb is a robust reagent for acute labeling of leukocytes during *in vivo* two-photon microscopy of the cortical microvasculature.

## 1 Introduction

Two-photon laser scanning microscopy is an advanced imaging approach that enables high-resolution imaging of the cerebral microvasculature in animal models.^1^ The activity of circulating immune cells is heavily regulated, and changes in this activity are an important indicator of disease. Increased leukocyte adhesion to the endothelium occurs in multiple neuroinflammatory conditions.^2^^,^^3^ While histology is a useful technique, the dynamics of leukocyte circulation and adhesion require *in vivo* observation.

Rhodamine 6G, a fluorescent dye, has been the standard marker for labeling leukocytes *in vivo* for two-photon imaging.^4^^,^^5^ However, rhodamine 6G labels both leukocytes and platelets, and additional markers are required to differentiate between these cell types. In addition, rhodamine 6G has a wide emission spectrum, which makes it difficult to use together with other fluorophores.^6^^,^^7^ There is a need for alternative leukocyte-specific markers that are accessible, long-lasting, and suitable for multichannel imaging experiments.

CD45.2 is an allele variant of the transmembrane protein tyrosine phosphatase receptor type C (PTPRC, also known as CD45). CD45.2 is encoded by the $Ptprc^{b}$ allele and is present on the surface of all mouse leukocytes except in strains bearing the CD45.1 ($Ptprc^{a}$) allele, such as SJL mice.^8^ The anti-mouse CD45.2 monoclonal antibody (clone 104) has been thoroughly validated as a murine pan-leukocyte marker for flow cytometry.^9^^,^^10^ Clone 104 does not bind the CD45.1 allele. The function of CD45 is complex and divergent between mice and humans, with multiple splicing variant isoforms involved in human T cell differentiation.^11^ Clinically, radioconjugates of anti-CD45 antibodies have been tested for bone marrow ablation for transplant preconditioning.^12^

To establish antibody dosage for optimized *in vivo* two-photon imaging of anti-CD45 labeled leukocytes, we first injected low, medium, and high concentrations of CD45.2 mAb intravenously (i.v.) and analyzed the fractions of labeled and saturated cells via flow cytometry. We then investigated potential for cell depletion and characterized its imaging parameters *in vivo* during two-photon microscopy. We show that CD45.2 mAb fluorescent conjugates are useful *in vivo* leukocyte marker for two-photon microscopy. Injection of CD45.2 mAb does not cause global leukocyte depletion. The antibody labels leukocytes specifically and with strong intensity ideal for observation of CD45.2+ cells deep within the cortex. We also demonstrate that CD45.2 mAb are useful for detection of pathological leukocyte-endothelial interactions during venous stroke.

## 2 Methods

### 2.1 Animals

Wild-type BALB/c mice were purchased from Jackson Labs (#000651; Bar Harbor, Maine). Male and female mice aged 2 to 4 months were used for all experiments. Mice were socially housed five or less per cage in standard cages on a 12-h light-dark cycle, and those with cranial window implants were housed singly. All mice were monitored daily during the experimental period. Experiments were approved by the Seattle Children’s Research Institute Animal Use and Care Committee.

### 2.2 *In Vivo* Antibody Injection

Anti-mouse CD45.2 antibodies conjugated to APC, FITC, or Alexa 594 (clone 104, cat # 109814, 109805, or 109850, respectively; Biolegend) were injected at concentrations of 0.04, 0.4, or 2  mg/kg in a total volume of 50 to 100  μL. Isotype control antibody (clone MOPC-173, cat # 400207; Biolegend) was used at 0.4  mg/kg. Antibodies were diluted in sterile saline and injected into the retro-orbital venous plexus.

### 2.3 Blood Analysis

**Fig. 1:**
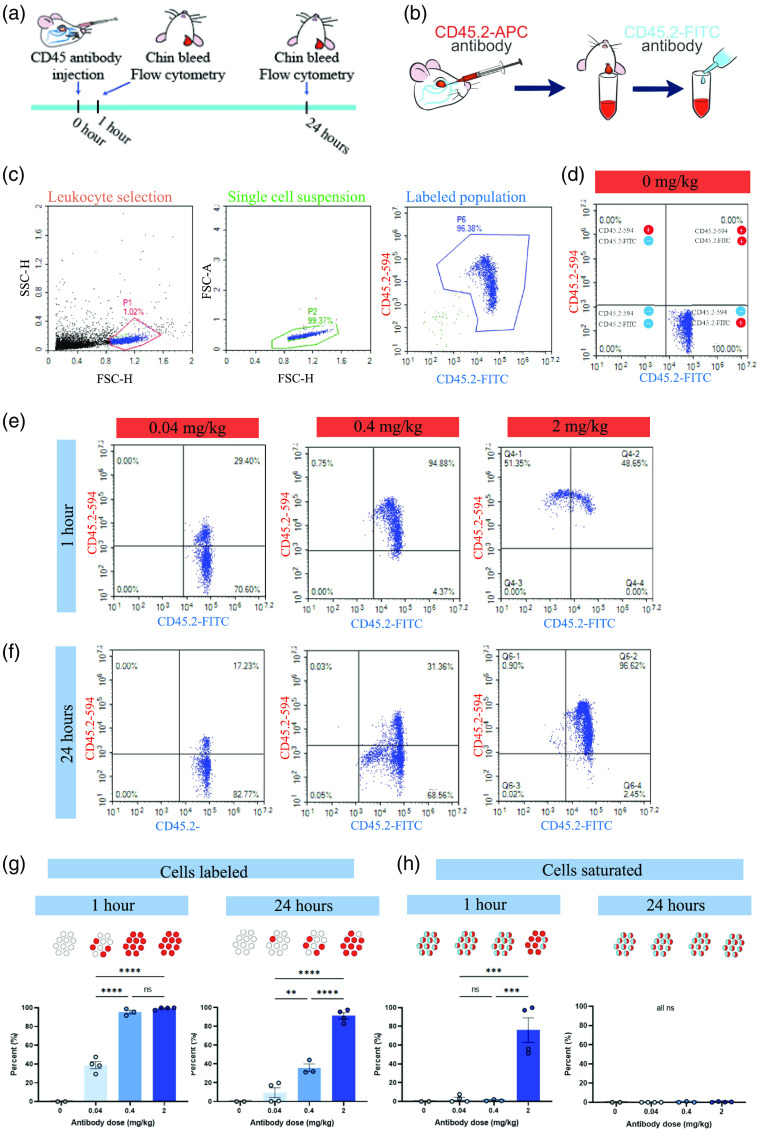
CD45.2 antibody labeling and saturation. (a) Schematic of experimental timeline. (b) Schematic of antibody labeling. (c) Flow cytometry gating strategy for 0.4  mg/kg i.v. antibody dose. Leukocytes were identified and cell debris excluded using the P1 gate. P2 denotes single cells. Finally, the P3 gate was used to identify leukocytes that were CD45.2-APC+ and/or CD45.2-FITC+. CD45.2 negative cells were omitted from analysis. (d)–(f) Examples of flow cytometry results, each plot shows representative data from one mouse. The x-axis is FITC antibody fluorescence intensity, and the y axis is APC antibody fluorescence intensity. Quadrants I, II, IV quantify double positive, CD45.2-APC+, and CD45.2-FITC+ leukocyte fractions, respectively. (d) Quantification of CD45.2 antibody labeling for 0  mg/kg i.v. antibody dose. (e) Quantification of CD45.2 antibody labeling after 1 h for 0.04, 0.4, and 2  mg/kg i.v. antibody doses. (f) Quantification of CD45.2 antibody labeling after 24 h for 0.04, 0.4, and 2 mg/kg i.v. antibody doses. (g) Quantification of leukocytes labeled with i.v. CD45.2-APC antibody after 1 h (comparisons not shown: 0 versus 0.04****, 0 versus 0.4****, 0 versus 2****) and 24 h (not shown: 0 versus $0.04^{\mathrm{ns}}$, 0 versus 0.4**, 0 versus 2****). (h) Quantification of cells where all CD45.2 sites are saturated with i.v. CD45.2-APC antibody after 1 h (comparisons not shown: 0 versus $0.04^{\mathrm{ns}}$, 0 versus $0.4^{\mathrm{ns}}$, 0 versus 2**) and 24 h (not shown: 0 versus $0.04^{\mathrm{ns}}$, 0 versus $0.4^{\mathrm{ns}}$, 0 versus $2^{\mathrm{ns}}$, 0.04 versus $0.4^{\mathrm{ns}}$, 0.04 versus $2^{\mathrm{ns}}$, 0.4 versus $2^{\mathrm{ns}}$). (g), (h) Each point represents data from one mouse (0  mg/kg n=2, 0.04  mg/kg n=4, 0.4  mg/kg n=3, 2  mg/kg n=4), bars show the mean and SEM, statistics by one-way ANOVA with Tukey correction for multiple comparisons between groups, ** P<0.01, *** P<0.001, **** P<0.0001, and ns denotes P≥0.05.

For flow cytometry analysis, blood was collected from the mouse facial vein by chin bleed 1 and 24 h after antibody injection [Fig. 1(a)]. Up to 50  μL of blood was collected with this method. For complete blood count (CBC), a terminal blood draw of 200 to 1000  μL was taken from the inferior vena cava 24 h after antibody injection. Collected blood was immediately transferred into tripotassium ethylenediaminetetraacetic acid (K3EDTA)-coated blood collection tubes (95057-291, Greiner Bio-One) and kept at 4°C until processing. CBC with differential counts of leukocyte subsets were measured on a Sysmex-XT-2000iV analyzer.

### 2.4 Cranial Window Surgery and Anesthesia

Thinned-skull windows were created over the right somatosensory cortex of mice as previously described.^1^^,^^13^ In brief, mice were induced with 4% isoflurane (07-893-8441, Patterson Veterinary) and maintained at 2% to 3% isoflurane for surgery. Analgesia was provided with 0.05  mg/kg buprenorphine. After removing the skin, an aluminum flange was secured to the skull for immobilization of the head during imaging. A ∼3-mm diameter region of the skull was thinned with a handheld drill until translucency. The area was dried and then covered with cyanoacrylate instant adhesive (Loctite 401) and a coverslip. Metabond dental cement (S380, Parkell) was then used to cover and secure the surgical preparation with the exception of the cranial window. Mice were given at least 24 h for recovery before imaging.

Acute skull-removed windows were created over the right somatosensory cortex of the brain.^14^ Similar to the thinned-skull window surgery, mice were given anesthesia and analgesic, the scalp was removed, and a head flange secured. A ∼3-mm circular outline was penciled on the skull. We then created a groove along the outline and removed the isolated cranial bone. A 5-mm coverslip was placed over the exposed brain. Then, a 1.5% agarose solution in sterile PBS was added to fill the space between the coverslip and brain for reduction of heartbeat- or breathing-related movement artifacts. Dental cement was used to seal the window. Mice were used immediately for imaging and euthanized within 24 h of cranial window surgery.

### 2.5 *In Vivo* Two-Photon Microscopy

Imaging was performed with a Bruker Investigator microscope with Prairie-View imaging software, coupled to a Spectra-Physics InSight X3 tunable laser (680 to 1300 nm) set to 800, 825, or 1100 nm excitation. Isoflurane at 4% and 1.5% to 2% was used to induce and maintain mice under anesthesia for imaging, respectively. Under 2% isoflurane, we injected 50  μL custom made 5% (w/v) 2 MDa Alexa 680-dextran^15^ retro-orbitally. A 4× (0.16 numerical aperture, NA) air objective (Olympus; UPLSAPO) was used to obtain an overview map of the window during the first imaging session. We then switched to a 20× (1.0 NA) water-immersion objective (Olympus; XLμMPLFLN) for high-resolution imaging. Alexa-594 fluorescently conjugated CD45.2 mAb and 70 kDa Texas Red-conjugated 70 kDa dextran intravascular dye (5% w/v in PBS, D1864; Fisher Scientific) signals were collected through the 595/50  nm bandpass filter; FITC-conjugated CD45.2 signals were collected through the 525/70  nm bandpass filter; Alexa 680-conjugated 70 kDa dextran emission was collected through a 660/40  nm bandpass filter.

### 2.6 Photothrombotic Venous Occlusions

Targeted photothrombotic occlusions of single venules were performed as previously described.^16^ A large cortical surface venule was occluded by irradiation with a focused green laser (532 nm). We injected 30  μL of 5% (w/v) Rose Bengal (330000-1G, Sigma-Aldrich) solution retro-orbitally. Photothrombotic occlusion was then induced by constant irradiation at 2 mW for 1 min. Z-stacks of the photothrombotic area were taken immediately before and after occlusion. Mice were then given 72 h to recover and monitored daily before the next imaging session.

### 2.7 Two-Photon Image Analysis

All image analysis was performed in ImageJ/Fiji. The Cell Counter plugin was used to assign an ID to each leukocyte and track cell movement throughout image stacks. Cells were classified as flowing if their velocity matched the red blood cell velocity within the vessel. Rolling and crawling cells were identified if cells were interacting with the vessel walls and slowed below the velocity of the surrounding RBCs. Finally, cells were classified as adherent if they had no movement for at least 30 min.

### 2.8 Flow Cytometry

Blood was collected in K3-EDTA tubes, red blood cells lysed using ammonium-chloride-potassium buffer, and lymphocytes collected via density gradient centrifugation in lymphocyte separation media. Leukocytes were then labeled with a saturating concentration of anti-mouse CD45.2-FITC antibody (clone 104, cat #109805, Biolegend) at 1:100 on ice and in the dark for 15 min and washed twice with autoMACS running buffer (cat #130-091-221, Miltenyi Biotec) before being analyzed on a Novocyte flow cytometer [Fig. 1(b)]. Unstained controls were used to exclude nonspecific fluorescence. The gating strategy consisted of selecting the leukocyte population and excluding cell debris, excluding doublets and cell clusters, and excluding CD45.2 negative cells [Fig. 1(c)]. The resulting population of CD45+ leukocytes was analyzed for colabeling of CD45.2-APC (injected *in vivo*) and CD45.2-FITC.

### 2.9 Statistical Analysis

GraphPad Prism 9 was used to perform all statistical analyses. Summary data of CBC and leukocyte subset counts as well as antibody saturation were expressed as mean ± SEM. Comparison between groups were made using a one-way ANOVA with Tukey correction for multiple comparisons. P≤0.05 was considered statistically significant.

To conduct power analysis for leukocyte depletion experiments, we performed a pilot study using the 0.4  mg/kg CD45.2 mAb dose (n=2) compared to isotype control (n=1). The difference in total white blood cell count between the groups (1800  cells/μL) was used to calculate Cohen’s d effect size. For an alpha of 0.05 and a power of 0.8, a cohort size of n=3 experimental animals and controls each was required to detect a difference of 1800  cells/μL.

## 3 Results

### 3.1 Anti-CD45.2 mAb Dose and Receptor Saturation

We first conducted experiments to determine the minimum dose required to label all circulating leukocytes *in vivo*. We intravenously injected APC-conjugated CD45.2 mAb (CD45.2-APC) at 0.04, 0.4, and 2  mg/kg doses. These doses were chosen to represent a low, medium, and high antibody concentration based on previous work using anti-Ly6G mAb leukocyte labeling for *in vivo* two-photon brain imaging.^17^ We then collected blood 1 h and 24 h postinjection [Fig. 1(a)]. Leukocytes were then counterstained *ex vivo* with a saturating concentration of FITC-conjugated CD45.2 mAb (CD45.2-FITC) to determine both the total number of leukocytes present and to assess what percentage of CD45.2 molecules were labeled by the i.v. CD45.2-APC antibody [Fig. 1(b)]. Antibody labeling and saturation were analyzed using flow cytometry with a gating strategy to exclude cell debris, select single cells, and finally isolate the positively labeled populations for analysis [Figs. 1(c)–1(f)].

One hour after CD45.2-APC antibody injection, large numbers of circulating cells were labeled [Fig. 1(g)]. The lowest injected dose, 0.04  mg/kg, marked 39% (SEM±3.7) of total leukocytes. The medium dose, 0.4  mg/kg, labeled 95% (SEM±2.0) of leukocytes. The highest antibody dose, 2  mg/kg, labeled nearly all leukocytes [99%, SEM±0.6; Fig. 1(g)]. The 24-h time point provided us with data on antibody persistence [Fig. 1(g)]. At 24 h, 9.5% (SEM±5.3), 36% (SEM±4.2), and 91% (SEM±3.3) of cells remained labeled at the low, medium, and high antibody doses, respectively [Fig. 1(g)].

CD45.2 binding epitope saturation by the i.v. injected CD45.2-APC was quantified by measuring the fraction of cells that had no more binding sites available for *ex vivo* labeling with CD45.2-FITC [Figs. 1(d)–1(f)]. The low and medium doses of CD45.2 mAb did not saturate the CD45.2 binding sites, and FITC fluorescent colabeling was found on nearly all cells [Fig. 1(h)]. At the highest antibody dose, however, 76% (SEM±13) of leukocytes had all binding sites saturated [Fig. 1(h)]. CD45.2 binding sites were no longer saturated 24 h after mAb injection [Fig. 1(h)].

### 3.2 Imaging Properties of CD45.2 mAb *In Vivo*

Based on our dose-finding experiments, we chose the medium dose of antibody, 0.4  mg/kg, for *in vivo* two-photon imaging, as this was the minimum sufficient dose to label almost all circulating leukocytes. We reasoned that minimizing the antibody dose would reduce the likelihood of target cell depletion or other unanticipated biologic effects.

Most of our imaging experiments were done on a thinned-skull window preparation due to the short recovery period and lack of significant post-surgical inflammation.^13^^,^^18^ Acute skull-removed cranial windows were used for deeper visualization of labeled leukocytes.^14^

**Fig. 2:**
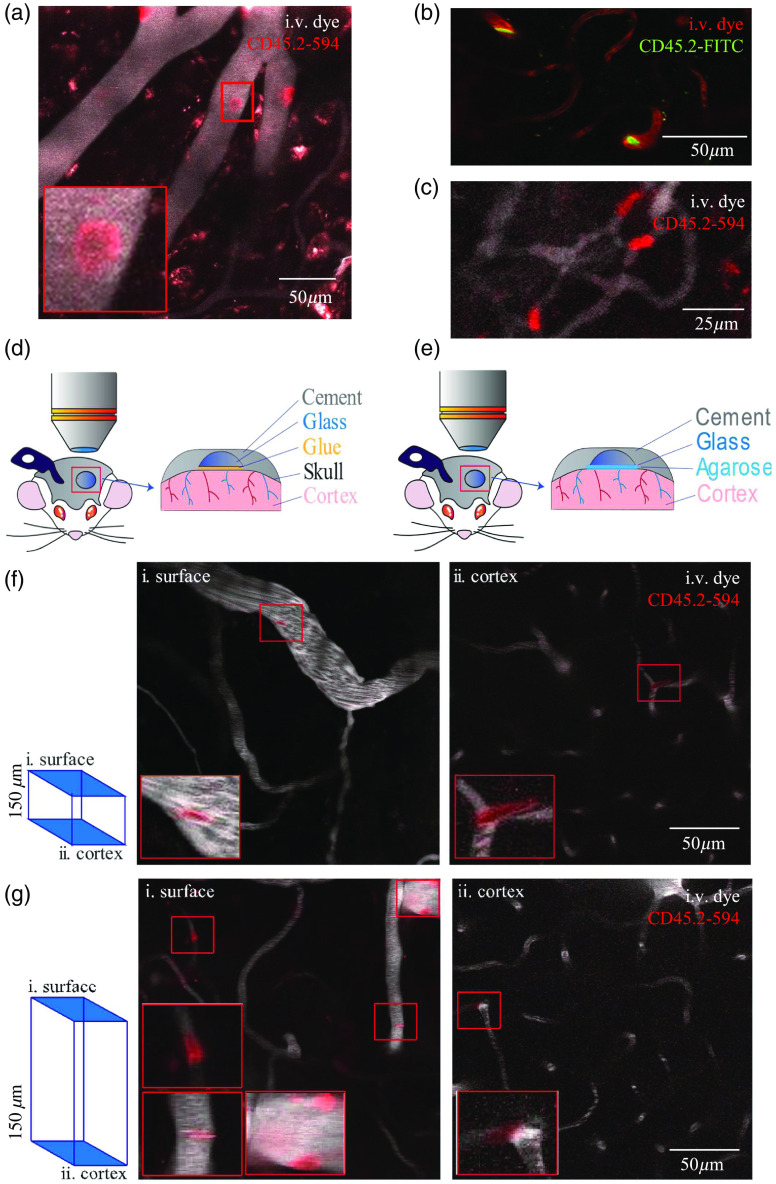
*In vivo* two-photon imaging of CD45.2 mAb labeling depth and persistence in the cerebral microvasculature. (a) Representative image of CD45.2-594 mAb cell labeling in superficial cortical microvessels. (b) Representative image of CD45.2-FITC mAb cells flowing through capillaries. (c) Representative image of leukocytes deforming to fit in capillaries. (d) Schematic of thinned-skull window *in vivo* preparation. (e) Schematic of acute skull-removed window *in vivo* preparation. (f) Representative images of CD45.2+ cells at the pial surface and 150  μm into the cortex imaged through the thinned-skull window. (g) Representative images of CD45.2+ cells at the pial surface and 550  μm into the cortex imaged through the skull-removed window. (a), (f), (g) Red squares indicate close up of CD45.2+ cells in the imaging field are shown in Videos 1, 2, and 3 (Video 1, 0.301 MB, MP4 [URL: https://doi.org/10.1117/1.NPh.9.3.031917.1], Video 2, 1.511 MB, MP4 [URL: https://doi.org/10.1117/1.NPh.9.3.031917.2], and Video 3, 5.36 MB, MP4 [URL: https://doi.org/10.1117/1.NPh.9.3.031917.3]).

A single retro-orbital injection of Alexa 594-conjugated CD45.2 mAb was given to mice. Within <5  mins post-injection, antibody binding was sufficient to clearly delineate leukocyte morphology [Fig. 2(a); Video 1]. While Alexa 680 intravascular dye facilitates double labeling experiments, it is not as easily available as some of the other dextran dyes such as FITC or Texas Red. An alternative combination of Texas Red i.v. dye and FITC-conjugated CD45.2 mAb also yielded brightly labeled leukocytes circulating in the cerebral vasculature [Fig. 2(b); Video 2].

Leukocytes moving through capillaries modified their shape to fit the small capillary lumen [Fig. 2(c)]. We also noted recurrent leukocyte flow through some capillaries while no leukocytes were observed in other capillaries (Video 3). With the thinned-skull window preparation, CD45.2+ cells were seen from the pial surface to 150  μm into the cortex [Figs. 2(d) and 2(f)]. In acute skull-removed window preparations, CD45.2+ cells were identifiable up to 550  μm into the cortex [Figs. 2(e) and 2(g)].

After the initial injection of CD45.2 mAb, some CD45.2-labeled cells could still be seen throughout the microcirculation upon reimaging at the same site 24 h later. However, 3 days after antibody injection, we saw no remaining circulating labeled leukocytes. When we gave a booster injection of 0.4  mg/kg CD45.2-Alexa 594, abundant CD45.2+ cells were once again labeled with the mAb.

### 3.3 Anti-CD45.2 mAb Shows No Evidence of Depleting Leukocytes after a Single Dose

**Fig. 3:**
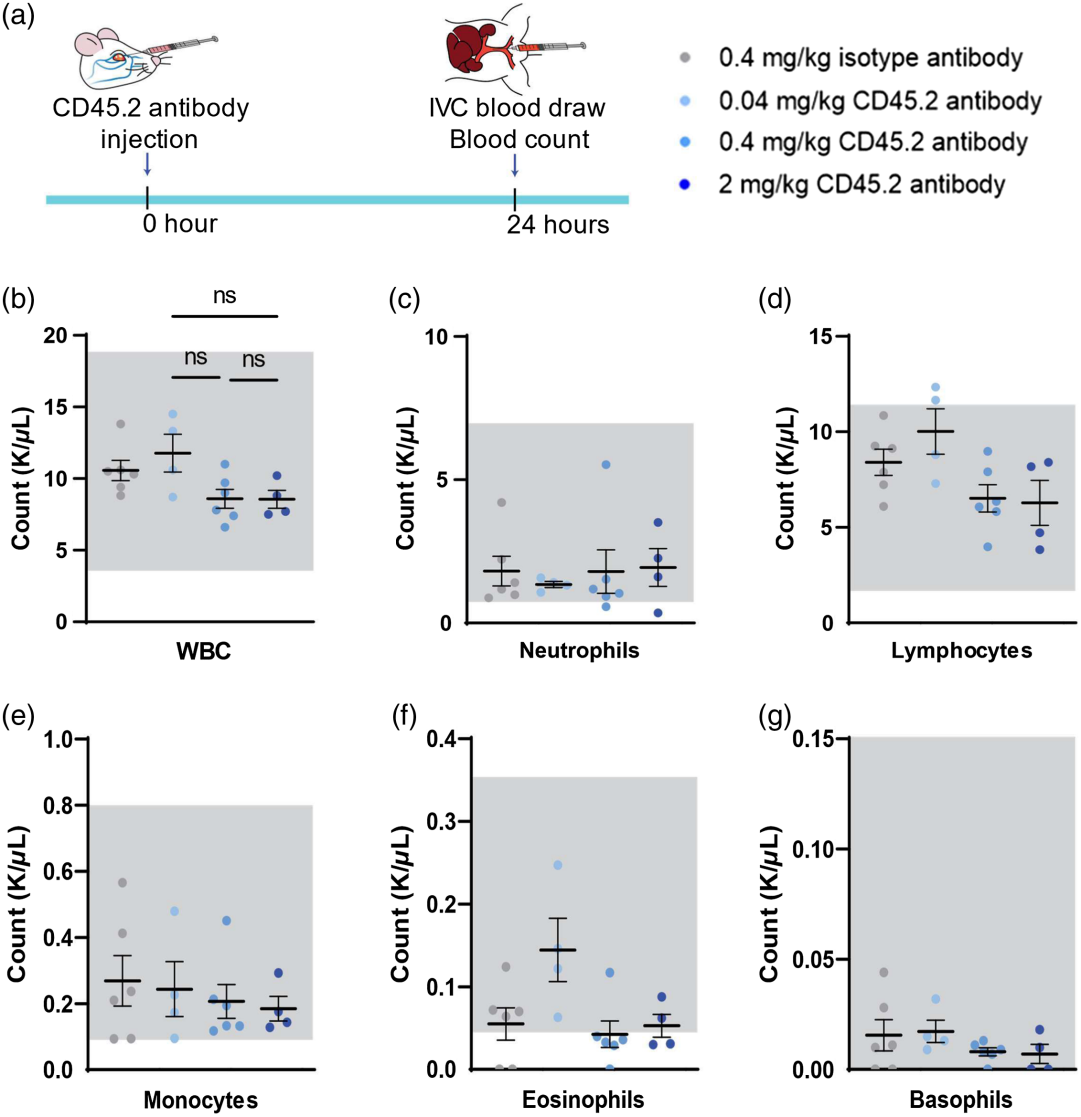
Absence of leukocyte depletion after a single CD45.2 mAb injection. (a) Schematic of the experimental timeline. (b) Quantification of cell counts of white blood cells in whole blood. Statistics performed by one-way ANOVA with Tukey correction for multiple comparisons between groups found all comparisons to be nonsignificant. (c)–(g) The differential cell type analyses of the samples shown in (b). (c) Quantification of neutrophils in whole blood. (d) Quantification of lymphocytes in whole blood. (e) Quantification of monocytes in whole blood. (f) Quantification of eosinophils in whole blood. (g) Quantification of basophils in whole blood. (b)–(g) Groups are ordered from left to right 0.4  mg/kg isotype antibody, 0.04  mg/kg CD45.2 mAb, 0.4  mg/kg CD45.2 mAb, and 2  mg/kg CD45.2 mAb; the gray boxes represent the normal range for each cell type;^19^ individual data points denote one animal (0  mg/kg n=6, 0.04  mg/kg n=4, 0.4  mg/kg n=6, 2  mg/kg n=4); y axis is the count of cells in 1000  cells/μl; bars represent the mean and SEM.

As monoclonal antibodies can induce ablation of target cells, we wanted to know if CD45.2 mAb affects the number of circulating leukocytes. Mice were given CD45.2 mAb antibody at 0.04, 0.4, and 2  mg/kg doses. A terminal blood draw was done 24 h after antibody injection [Fig. 3(a)]. CBC showed no evidence of leukocyte depletion and counts of leukocyte subsets were within the expected ranges with a few outliers (Fig. 3).^19^ The variability in CBC results among animals is not unexpected, as previous work has shown similar heterogeneity.^19^

### 3.4 *In Vivo* CD45.2 mAb in a Photothrombotic Occlusion Model

**Fig. 4:**
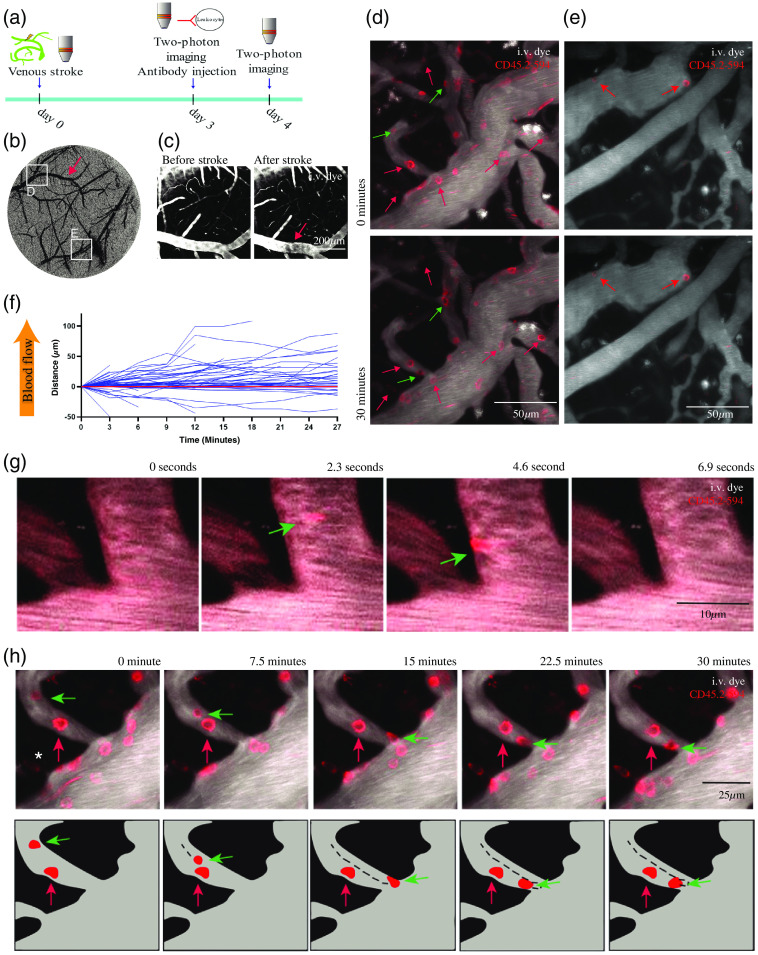
CD45.2 mAb application in a microstroke model. (a) Schematic of experimental timeline. (b) Thinned-skull window overview of the cortical surface vasculature. Location of panels (d) and (e) are on the window are marked with white squares. (c) Representative images of vein in the overview before and immediately after photothrombotic stroke. (b), (c) Red arrows denote irradiation area. (d), (e) *In vivo* two-photon images (z-projected image stack) showing the positions of adherent and crawling leukocytes at 0 and 30 min in a pial venule proximal to the area of occlusion (d) and in a location remote from the stroke (e). Red arrows denote adherent leukocytes that remained stationary for 30 min. Green arrows denote crawling leukocytes. (f) Movement of crawling and adherent leukocytes over 30 min (n=62 leukocytes). The positive direction on the y axis is the direction of blood flow. Images were obtained every 3 min. (g) Representative images of rolling cells, the acquisition frame period was 2.3 s. (h) Representative images of crawling and adherent cells denoted by green and red arrows, respectively. Z-stacks were acquired every 7.5 min. An asterisk is used to indicate an out of focus intravascular leukocyte. The irregular areas of hazy red signal represent autofluorescence. The schematic below the panel shows only the two highlighted cells for better visualization of movement.

To test the application of the CD45.2 antibody in a proinflammatory condition, we performed *in vivo* cell labeling experiments after a photothrombotically induced venous microstroke. Photothrombotic occlusions were performed by irradiating a large cortical surface vein after i.v. injection of Rose Bengal [Figs. 4(a)–4(c)]. Immediately after the stroke, venous flow became sluggish. In the following 48 h, stroke-induced dye extravasation obscured imaging of the vasculature, as background fluorescence intensity was similar to that of intravascular dye. We thus waited 72 h after the stroke to test leukocyte labeling because this was the earliest time point with imaging clarity. Within <5  min after injecting 0.4  mg/kg CD45.2-Alexa 594, we observed strong labeling of leukocytes [Fig. 4(d)]. Labeled leukocytes exhibited increased adherence to the endothelium in the venules and capillaries immediately surrounding the stroke compared to leukocytes in vessel segments further away [Figs. 4(d) and 4(e)].

We then used this photothrombotic occlusion model to test whether antibody-mediated cell labeling is stable enough to measure leukocyte dynamics over time. We noted four distinct movement types: flowing, rolling, crawling, and adherent. Flowing leukocytes were typically captured in distorted shapes due to limitations in scanning speed, moving at the same speed as blood flow and often present in a single imaging plane for only 0.5 to 1 s. Rolling leukocytes, defined as cells moving slower but in the direction of blood flow, were visible in a single imaging plane anywhere from 2.3 to 20 s [Fig. 4(g)]. Movements of crawling leukocytes, unlike that of flowing or rolling cells, were observed on a longer time scale [Figs. 4(f) and 4(h)]. When examining image stacks of venous flow that were acquired over 30 min, we saw crawling leukocytes displace 49±29  μm (mean±SD) from the starting position along the luminal surface of the endothelial cells [Fig. 4(f)]. Crawling leukocytes moved both with and against the direction of blood flow, although the overall final displacement favored movement with the blood flow [Fig. 4(f)]. Within the same image stacks, we also observed cells that remained in the same location for 30 min [Figs. 4(d), 4(e), and 4(h)]. However, it is important to note that we did not observe any leukocytes extravasating during imaging, and no extravascular CD45.2 labeled leukocytes were seen at any timepoint including 72 h post-stroke.

## 4 Discussion

In this study, we validated an alternative tool to label leukocytes for prolonged two-photon imaging experiments. We show that the CD45.2 mAb can be used *in vivo* to target a highly expressed pan-leukocyte cell surface marker that excludes platelets^9^^,^^10^ Targeting CD45 with fluorescent-conjugated antibody for *in vivo* two-photon imaging enables clear visualization of leukocyte movement and morphology, and we noted no loss of labeled cells within 30-min imaging experiments. The wide availability of the clone 104 mAb with a variety of conjugated fluorophores makes it an easy-to-use tool that can be incorporated flexibly into multicolor imaging experiments. It complements previously validated tools such as the Ly6G mAb for *in vivo* neutrophil labeling,^17^ and transgenic reporter mouse lines for leukocyte subsets such as Nr4a1-GFP, CD11c-YFP, or Cx3cr1-EGFP.^20^

We optimized the antibody dose at 0.4  mg/kg as the smallest necessary dose to label almost all circulating leukocytes and minimize the potential for unanticipated biologic effects. Higher doses may be helpful to improve imaging brightness and depth of observation. At the chosen imaging dose of 0.4  mg/kg, the fraction of labeled leukocytes decreased from 95% to 36% within 24 h, even though peripheral leukocyte counts remained stable within this timeframe. No labeled leukocytes were circulating after 72 h. A booster dose was required to restore leukocyte labeling at the later time point. The observed labeling reduction could be due to receptor turnover, physiologic cell turnover, or possibly antibody-induced ablation.^21^^,^^22^ Existing literature on leukocyte turnover does not completely explain why we no longer saw labeled leukocytes 72 h after mAb injection. The leukocyte pool in the peripheral circulation, chiefly composed of neutrophils and lymphocytes, is expected to persist from several hours for neutrophils and monocytes, to months for lymphocytes.^19^^,^^22^ While CD45.2+ neutrophils may have been turned over at 72 h, CD45.2+ lymphocytes should still be present in the circulation. This suggests that, despite our data demonstrating a lack of significant loss of any leukocyte subsets after 24 h, there may still be an element of ablation of labeled cells or unbinding of the antibody from labeled leukocytes. Removal of free antibodies from the plasma over time means that newly generated leukocytes will be labeled more dimly or not at all, but reduction of CD45.2 expression cannot be excluded.^23^ For practical imaging purposes, additional injections of CD45.2 mAb can be used to restore leukocyte labeling on successive imaging experiments.

We demonstrated the utility of CD45.2 mAb in a microstroke model, where ischemic injury is followed by rolling and adhesion of leukocytes.^24^^,^^25^ In our imaging experiments, we observed many cells that were rolling at various speeds. These were distinct from other cells that appeared stationary on initial inspection and only over many minutes crawled slowly along the luminal side of the endothelium. A consensus on classification of rolling, crawling, and adherent cells *in vivo* is yet to be determined. While other work studying *in vivo* leukocytes defined adherent cells as cells that remained stationary for 30 s or longer, we found some of these cells to be crawling intermittently.^26^

We noted adherent cells that remained stationary for the entire 30-min duration of the experiment. Nonetheless, we did not observe leukocyte extravasation during any of our experiments despite a prolonged period of observation, which should have been sufficient to capture this phenomenon.^27^^,^^28^ The small size and rapid recanalization of the occluded venule in our microstroke model may have caused insufficient cellular damage to stimulate leukocyte extravasation. Leukocyte extravasation could also have occurred outside of our observation period or area of observation. It appears unlikely that the CD45 label could be lost during extravasation. CD45.2 receptors are not known to interact with the endothelium,^29^ and indeed, cells that were adherent to the endothelium immediately after antibody injection were not dislodged after 30 min of imaging.

In conclusion, the CD45.2 mAb leukocyte labeling method for *in vivo* two-photon brain imaging is promising as a flexible tool that can be employed in various murine disease models to characterize leukocyte behavior.
